# Modular architecture and resilience of structural covariance networks in first-episode antipsychotic-naive psychoses

**DOI:** 10.1038/s41598-023-34210-y

**Published:** 2023-05-12

**Authors:** Madison Lewis, Tales Santini, Nicholas Theis, Brendan Muldoon, Katherine Dash, Jonathan Rubin, Matcheri Keshavan, Konasale Prasad

**Affiliations:** 1 Department of Bioengineering, Swanson School of Engineering, University of Pittsburgh, 3811 O’Hara St, Pittsburgh, PA 15213 USA; 2 Department of Psychiatry, University of Pittsburgh School of Medicine, Pittsburgh, PA 15213 USA; 3 Department of Mathematics, University of Pittsburgh, Pittsburgh, PA 15213 USA; 4 Department of Psychiatry, Beth Israel Deaconess Medical Center, Harvard Medical School, Boston, MA 02215 USA; 5 Veterans Affairs Pittsburgh Health System, University Drive, Pittsburgh, PA 15240 USA

**Keywords:** Neuroscience, Medical research, Engineering

## Abstract

Structural covariance network (SCN) studies on first-episode antipsychotic-naïve psychosis (FEAP) have examined less granular parcellations on one morphometric feature reporting lower network resilience among other findings. We examined SCNs of volume, cortical thickness, and surface area using the Human Connectome Project atlas-based parcellation (n = 358 regions) from 79 FEAP and 68 controls to comprehensively characterize the networks using a descriptive and perturbational network neuroscience approach. Using graph theoretical methods, we examined network integration, segregation, centrality, community structure, and hub distribution across the small-worldness threshold range and correlated them with psychopathology severity. We used simulated nodal “attacks” (removal of nodes and all their edges) to investigate network resilience, calculated *DeltaCon* similarity scores, and contrasted the removed nodes to characterize the impact of simulated attacks. Compared to controls, FEAP SCN showed higher betweenness centrality (BC) and lower degree in all three morphometric features and disintegrated with fewer attacks with no change in global efficiency. SCNs showed higher similarity score at the first point of disintegration with ≈ 54% top-ranked BC nodes attacked. FEAP communities consisted of fewer prefrontal, auditory and visual regions. Lower BC, and higher clustering and degree, were associated with greater positive and negative symptom severity. Negative symptoms required twice the changes in these metrics. Globally sparse but locally dense network with more nodes of higher centrality in FEAP could result in higher communication cost compared to controls. FEAP network disintegration with fewer attacks suggests lower resilience without impacting efficiency. Greater network disarray underlying negative symptom severity possibly explains the therapeutic challenge.

## Introduction

Schizophrenia is associated with widespread alterations in gray matter volume (GMV), cortical thickness (CT), and surface area (SA), but the findings are inconsistent^1,2^. Besides medications and illness heterogeneity, such inconsistencies may be due to altered morphometry within a network of regions with different degrees of covariance resulting in a few regions showing group differences depending on the degree of covariance. Such covariance patterns can be better captured in structural covariance networks (SCNs) that are quantitative mathematical representations of shared regional morphometric variations. Group-level SCNs reduce a complex system (i.e., group-wide morphometric alteration patterns) to an abstract structure of a correlation matrix highlighting between-subject differences in covariance patterns. This is supported by observations of between-subject variability of regional volumes being greater than between-subject differences in whole brain volume and by between-subject differences in one region covarying with between-subject differences in other regions^3,4^. These observations are hypothesized to be due to underlying structural^5,6^ and functional^7,8^ connectivity. Such covariance patterns have revealed global and regional structural ‘connectivity’ differences in schizophrenia compared to controls^9^ that may start as early as in neonatal infants at familial high-risk for schizophrenia^10^ associated with specific cognitive networks^9,11^. Further, since the white matter tracts partly correspond to the SCN edges^12^, SCN connectivity partly reflects the underlying anatomical connections.

Three studies have investigated the SCN of first-episode schizophrenia using graph theoretic methods via a descriptive network neuroscience approach. One study using group-level weighted SCN of GMV reported higher degree nodes in a combined sample of first-episode (but not antipsychotic-naïve) and chronic patients compared to healthy controls (HC) but first-episode and chronic patients did not differ^13^. Another study of group-level SCN of local gyrification index 68 regions-of-interst (ROIs) among unmedicated patients reported no differences in path length and global efficiency over 6 weeks^14^. An investigation of group-level SCN of CT reported significant covariance difference with HC in the subnetwork comprised of temporal and frontal regions of first-episode schizophrenia patients, but not among chronic or treatment-resistant schizophrenia patients^15^. These studies used atlases based on anatomical landmarks or cytoarchitectonic patterns that did not include structural or functional connectivity, e.g., Desikan-Killiany-Tourville (DKT)^13,14^ and the Destrieux atlas^15^, and examined one morphometric measure of regions, such as GMV or CT that does not provide a composite picture of morphometric variations as we highlighted in our systematic reviews^16,17^.

A perturbational network neuroscience approach involves investigating the impact of simulated manipulation of components of a network, e.g., nodes and edges, to gain a deeper understanding of the network during the progression of pathophysiology, disease, or treatment. One study on gyrification SCN among chronic schizophrenia patients reported reduced resilience of schizophrenia network^18^. Another study using resting fMRI reported no significant difference in the resilience of functional network in childhood-onset schizophrenia^19^. Perturbational neuroscience approach to test the resilience of SCN of first-episode antipsychotic-naïve psychosis (FEAP) patients may reveal critical nodes responsible for longitudinal changes during disease progression since increasing structural covariance strength with disease progression has been reported^13^. We tested the resilience comprehensively by using “attack” simulations by targeted and random removal of nodes and all their edges followed by sequentially evaluating giant connected component (GCC) and network similarity using the *DeltaCon* similarity score^20^.

We used the Human Connectome Project (HCP) Multi-Modal Atlas (version 1)^21^ which parcellates the cortex into 358 functionally and structurally connected, and cytoarchitecturally defined regions, allowing for better interpretations of regional covariance structure and highlighting SCN connectivity of anatomically smaller regions that may be pathophysiologically significant. Such an approach is supported a prior study that did not find differences in an SCN consisting of whole thalamic volume and cortical Brodmann areas between schizophrenia and controls^22^ but in an SCN of volumes of thalamic nuclei and cortical Brodmann areas, patients showed correlation of pulvinar with frontal cortical Brodmann areas while controls showed correlation of centromedian nucleus with cortical Brodmann areas^23^. We built group-level SCN using GMV, CT and SA to investigate differences in the covariance pattern of morphometric features between the groups since it allowed us to control for age, sex and total GMV, total SA and mean CT, which is not possible when individual networks of morphometric covariance are built. An individual SCN has the advantage of providing graph metrics for each subject that can be controlled for the covariates after the graph analysis. However, our preference was to control for covariates while building the network. Further, investigating modularity index using the Newman method would be challenging on subject-level graphs due to the arbitrary module numberings^24^.

Our goal was to examine characteristics of SCNs using measures of segregation, integration, centrality, resilience, community structure, and network hubs. We examined FEAP patients to minimize the impact of medication use and illness chronicity. Based on previous studies that reported higher degree nodes^13,25^, higher number of hubs^26^, and decreased resilience^18^, we hypothesized that the FEAP SCN will be less resilient compared to controls. Specifically, we predicted that consecutive attacks in a descending order of node centrality would result in the remaining nodes in the SCN of patients disintegrating with removal of fewer nodes compared to that of controls with sufficient node removals. Network disintegration was defined as a decrease in the GCC by a moving average of ≥5 nodes with the removal of one node in the preceding step. Further, we predicted that the SCN of FEAP and HC would show low similarity score before the attack that would increase with the removal of high impact nodes.

## Methods

### Subject recruitment

We enrolled 84 FEAP patients of both sexes between the ages 12 and 50 years from inpatient and outpatient facilities of the Western Psychiatric Institute and Clinic, Pittsburgh, and 71 HC from the same neighbourhoods as the patients. We excluded individuals with intellectual disability, substance dependence within the past 6 months and/or abuse in the month preceding enrollment per DSM-IV, significant medical/neurological disorders, and prior antipsychotic treatment^27^. Consensus diagnosis was made by senior clinicians after reviewing the Structured Clinical Interview for DSM IV (SCID-IV), medical records, and 6-month follow-up information. The study was approved by the University of Pittsburgh Institutional Review Board and all methods used in this study conform to the approved protocol in accordance with the relevant guidelines and regulations. After complete description of the study, informed consent was obtained from all subjects. Severity of psychopathology was evaluated using the Scale for Assessment of Positive Symptoms (SAPS)^28^ and the Scale for Assessment of Negative Symptoms (SANS)^29^.

### Imaging methods

Details of MRI scanning are published^27^. Briefly, T1-weighted 3-dimensional spoiled-gradient-recalled (3D-SPGR) MRI data was acquired on a 1.5 T GE whole-body scanner (124 contiguous coronal slices perpendicular to the anterior commissure-posterior commissure line, 1.5-mm thickness, steady-state pulse sequence: TE = 5 ms, TR = 25 ms, matrix = 256 × 192, FOV = 24 cm and flip angle = 40°).

Using FSL 6.0, the images were motion and bias distortion-corrected, skull-stripped, and then visually inspected for optimum segmentation and quality. The T1-weighted images were initially processed with FreeSurfer 6.0, mapping each subject into fsaverage space. The subject-level HCP parcellations were then created from the fsaverage HCP annotation file^30^, and mapped to native space of each individual using the Neurolab^31^. From these parcellations, we extracted the GMV, SA, and CT for each HCP region. Good quality parcellation was checked manually. Five patients and 3 controls were excluded for poor scan quality (n = 7) and poor parcellation (n = 1).

We compared morphometric measures of brain regions separately between FEAP and HC using MANCOVA by including total GMV, mean CT and total SA respectively for each morphometric type and age and sex for all morphometric comparisons to elucidate quantitative changes in these measures followed by Bonferroni-corrected between-subjects’ effects. Schizophrenia (schizophrenia, schizoaffective disorder, and schizophreniform disorder diagnoses) and and non-schizophrenia groups were compared with HC using the same approach.

### SCN analysis

Using an in-house developed code writen in MATLAB (version R2019a), separate SCNs consisting of GMV, SA, and CT of group-level HCP parcels (nodes) were built using partial correlations (edges) across all subjects in each diagnositc group controlling for total brain GMV, total SA, or average CT respectively. Age and sex were additional covariates common for networks of all three morphometries. The adjacency matrices of the SCNs represented group-level regional covariance patterns. We first compared the SCN of FEAP subjects with SCN of HC followed by post hoc comparison of SCN of schizophrenia and SCN of non-schizophrenia groups with SCN of controls. Two-hundred random graphs were constructed by randomizing each group SCN while preserving degree distribution^32^. With regard to number of randomizations, the literature recommendations vary from 100 to 1000. We randomized until we obtained stable clustering coefficient (CC) and characteristic pathlength, which was 200 for our data. We used the absolute value of correlations instead of positive or negative correlations and examined undirected SCNs. Both the network density and edge-weight intensity thresholding methods can be used to minimize noise or unlikely network connections; an intensity threshold removes any edge with an edge weight below the chosen threshold value, while a density threshold removes the lowest weight edge until a chosen network desity is achieved. To allow variability in network density so that both hypo- and hyperconnectivity may be observed, which is not possible with density threshold^33^, we used a partial correlation intensity threshold range based on small worldness ($\upsigma$):

$${\upsigma } = \frac{{{\raise0.7ex\hbox{$C$} \!\mathord{\left/ {\vphantom {C {C_{R} }}}\right.\kern-0pt} \!\lower0.7ex\hbox{${C_{R} }$}}}}{{{\raise0.7ex\hbox{$L$} \!\mathord{\left/ {\vphantom {L {L_{R} }}}\right.\kern-0pt} \!\lower0.7ex\hbox{${L_{R} }$}}}}$$

where *C* is the CC and *L* is the pathlength for each group SCN, ${C}_{R}$ and ${L}_{R}$ are the CC and pathlength, respectively of the random network^25^. To ensure that the SCNs of FEAP and HC were nonrandom, we analyzed over σ threshold range which compared the CC and the pathlength of the SCN with the random SCN. The lowest partial *r* threshold corresponded to σ threshold at which all SCNs were nonrandom (σ ≥ 1.2) and the maximum partial *r* threshold was when all SCNs were connected graphs (graphs with a path between every pair of nodes, determined by the reachability matrix), where higher than the maximum threshold would yeild unconnected components. After thresholding, the networks were binarized to sample the graph metrics at reasonable threshold intervals to capture variablity at different threshold intervals.

Within the experimentally determined threshold range (intensity threshold, partial *r* = 0.075–0.275), we calculated the graph metrics representing integration (characteristic pathlength and eccentricity), segregation (CC and modularity), centrality (degree, betweenness centrality (BC), and eigenvector centrality (EC)), and assortativity at the smallest threshold intervals (0.025) at which smoother variation across the σ threshold ranges can be extracted to capture variabilities of the graph metrics and obtain a reasonable number of samples to statistically compare the groups. The graph metrics were calculated from the SCNs of each group for each morphometric measure using the Brain Connectivity Toolbox^33^. A brief overview for these measures has been provided in Supplemental Table 1 and in our prior publication^16^. Modularity, characteristic pathlength, and assortativity were calculated as global measures across the σ range and other graph metreics as nodal measures averaged across all nodes at the same intervals. Group differences were examined for nodal meaures using t-tests at each threshold to compare graph metrics across nodes correcting for multiple tests using Bonferonni approach (9 intervals * 5 nodal graph metrics = 45 tests; critical α = 0.05/45 = 0.001). Global measures were examined across the σ range using a t-test. For global measures, a permutation test was done to ensure that nonrandom *p* values do not influence rejection/acceptance of the null hypothesis by randomizing groups 100 times, when the p-values stabilized in the randomization data, and comparing the real comparison p-value to the mean p-value from the randomized comparisons^18^. BC, CC, and degree were compared between groups for regions showing significant morphometric difference to investigate network architecture (segregation and centrality) in these regions.

**Table 1:** Nodal and Global graph measures averaged across entire threshold range for each group for the three morphometric features.

Average graph measure across threshold range	Volume SCN				Cortical thickness SCN				Surface area SCN			
	FEAP	SZ	nSZ	HC	FEAP	SZ	nSZ	HC	FEAP	SZ	nSZ	HC
Global SCN metric												
Characteristic Pathlength	1.86 ± 0.34	1.65 ± 0.20	1.59 ± 0.19	1.79 ± 0.28	1.81 ± 0.30	1.62 ± 0.19	1.58 ± 0.18	1.75 ± 0.25	1.94 ± 0.43	1.68 ± 0.20	1.59 ± 0.19	1.86 ± 0.35
Modularity	0.18 ± 0.11	0.11 ± 0.06	0.09 ± 0.05	0.15 ± 0.09	0.17 ± 0.12	0.10 ± 0.06	0.09 ± 0.04	0.14 ± 0.09	0.19 ± 0.12	0.12 ± 0.06	0.10 ± 0.05	0.17 ± 0.10
Assortativity	0.10 ± 0.07	0.11 ± 0.07	0.05 ± 0.04	0.10 ± 0.07	0.13 ± 0.1	0.06 ± 0.04	0.08 ± 0.06	0.07 ± 0.04	0.09 ± 0.06	0.14 ± 0.08	0.08 ± 0.05	0.13 ± 0.09
Nodal SCN metric												
Degree	87.5 ± 65.3	128.7 ± 68.4	148.3 ± 66.5	96.4 ± 67.3	79.7 ± 64.8	116.6 ± 69.9	146.5 ± 66.5	87.0 ± 67.0	94.3 ± 65.5	136.5 ± 67.4	150.8 ± 66.0	103.9 ± 66.5
Clustering coefficient	0.37 ± 0.11	0.43 ± 0.14	0.46 ± 0.15	0.37 ± 0.12	0.35 ± 0.11	0.39 ± 0.15	0.46 ± 0.15	0.34 ± 0.12	0.40 ± 0.10	0.46 ± 0.13	0.48 ± 0.14	0.41 ± 0.11
Betweenness centrality	306.7 ± 120.0	230.3 ± 69.9	209.7 ± 66.5	282.7 ± 99.8	336.6 ± 153.5	244.3 ± 73.2	211.5 ± 66.5	308.5 ± 125.0	290.7 ± 105.5	214.7 ± 68.4	238.1 ± 66.0	279.9 ± 88.8
Eccentricity	2.5 ± 0.66	2.15 ± 0.32	2.01 ± 0.02	2.35 ± 0.49	2.61 ± 0.78	2.19 ± 0.38	2.02 ± 0.05	2.48 ± 0.66	2.42 ± 0.53	2.13 ± 0.28	2.01 ± 0.03	2.34 ± 0.48

**Figure 1:**
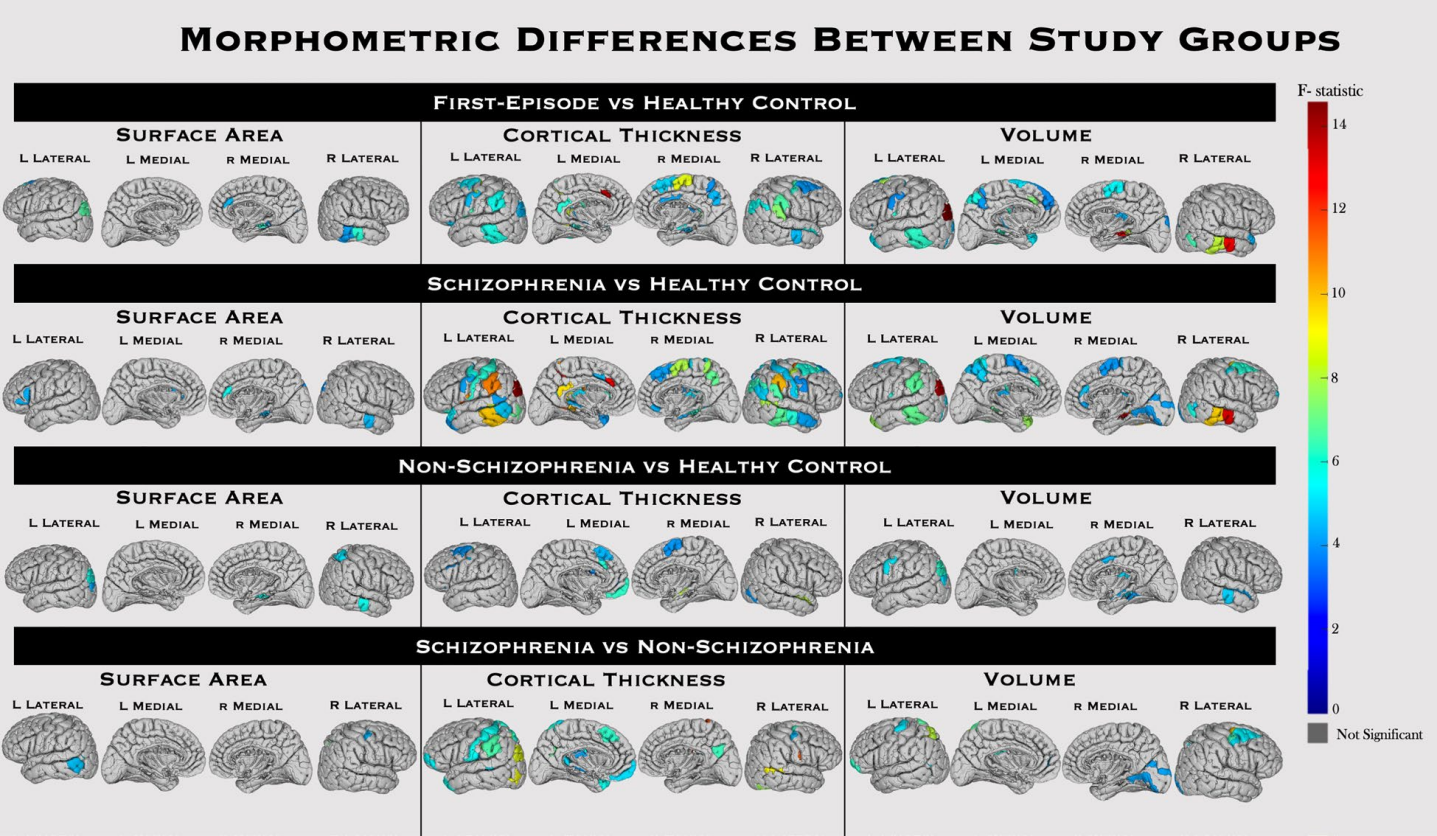
Morphometric Group Differences Throughout the Brain Regions*.* Color of regions represents F-statistic for the MANCOVA preformed to test group differences in all three morphometrics when comparing FEAP v HC (top), schizophrenia vs HC (top middle), non-schizophrenia vs HC (bottom middle), and schizophrenia vs non-schizophrenia (bottom). Regions that were not significant after Bonferroni correction are colored gray. See Supplemental Material Table 1 for all the significant region names and statistics.

Mean ± standard deviation is reported. See Fig. 1 or Supplemental Table 4 for variation in significantly different measures at each threshold interval within the small-worldness threshold range.

*FEAP* first-episode antipsychotic-naïve patients, *SZ* schizophrenia, *NSZ* non-schizophrenia, *HC* healthy control, *SCN* structural covariance network.

We next examined the community structure, and identified hubs over the σ range at intervals of 0.005 by maximizing modularity across the σ threshold range using the Louvain algorithm^34^ keeping the default resolution of γ = 1. Hubs were defined as nodes with degree, BC, or EC > 2 standard deviations of the same measure compared to network average^25^, and also found in ≥ 50% of the thresholded networks.

*Network resilience* was examined using simulated “attacks” (removal of nodes and all their edges) with and without replacement in the SCNs of GMV, CT, and SA across the σ threshold range at 0.025 intervals. First, individual nodes were sequentially attacked in the descending order of BC and EC until all nodes are removed (targeted cumulative attack). We also implemented two non-cumulative attacks where the attacked node was placed back before the next node was attacked. One involved attacking one randomly selected node at a time (random non-cumulative attack) and, the second one involved attacking in the descending order of BC (sequential non-cumulative attack). We chose BC and EC to rank the nodes for attacking because they determined the hubs and attacking them would ensure that the nodes with the greatest impact on the network are removed along with their edges. Degree-based attacks were not implemented because all degree-based hubs were in the centrality-based simulations. The GCC, which indicates the size of the largest set of mutually interconnected regions in the network scaled relative to the full network size, and the global efficiency were measured following each attack.

The *DeltaCon* similarity score^20^ was calculated before the attack and at the point where FEAP SCN first showed disintegration. The *DeltaCon* similarity score^20^ exhibits the properties of edge importance (changes leading to disconnected graph penalized more than the ones that maintain connectivity), weight awareness (the larger the weight of the removed edge, the greater the impact on similarity score), edge- “submodularity” (a specific change in sparse network is more important than in a much denser equally sized network) and focus awareness (random changes are less important than targeted changes of the same extent). The similarity score varies between 0 and 1, where 0 means totally different graphs and 1 means identical graphs, and is more robust than other network comparison metrics^35^.

### Association of graph measures with severity of psychopathology

Since the group-level SCN does not provide graph metrics for individual subjects, a median split of SANS and SAPS scores of FEAP patients was performed and SCNs for FEAP scoring above and below the median for SANS and SAPS scores were built, after which we calculated the graph measures across the threshold range, an approach used in a prior publication^18^. These four SCNs were compared with full HC SCN to obtain graph metrics across the threshold range applying the Bonferroni corrections. The relationship of graph metrics of SCNs of FEAP scoring above and below the median with severity of psychopathology was tested using *t*-tests.

### Conference presentation

A version of this data was presented at the 74th Annual Meeting of the Society of Biological Psychiatry held on May 16 to 18, 2019 at Chicago, IL.

## Results

### Clinical and demographic

The final sample of 79 FEAP consisted of schizophrenia (n = 41), schizoaffective disorder (n = 5), schizophreniform disorder (n = 1), delusional disorder (n = 2), psychotic disorder not otherwise specified (n = 12), bipolar disorders (n = 4), depressive disorders (n = 13), and unspecified mood disorders (n = 1) per DSM-IV. Sixty-eight controls met our MRI quality standards. Mean age and sex (Supplemental Table 2) of FEAP (23.99 ± 7.21 years; 56 males) and controls (24.59 ± 6.64 years; 39 males) did not differ (t = 0.52, p = 0.60; χ^2^ = 2.93, p = 0.09). Mean illness duration from the time of first psychotic symptom to MRI was 2.57 ± 3.35 years.

**Table 2:** Hubs identified for all networks*.*

Cortical thickness SCN			
Schizophrenia	Non-schizophrenia	FEAP	Healthy control
L Fourth Visual Area	L Perirhinal Ectorhinal Cortex*	R Area 1	R Area dorsal 32
L Middle Temporal Area	R Area TemporoParietoOccipital Junction 2	R Area 10r^	R Area a24
L Area 6 m anterior	R Superior Frontal Language Area	L Second Visual Area^	L Perirhinal Ectorhinal Cortex *
R Primary Motor Cortex	L Second Visual Area^	R Second Visual Area	L Area Lateral Occipital 2
R Area 9 Posterior	R Area anterior 10p	R Area 8BM	L Area TG Ventral
L Ventral Area 6	R Area PF opercular	R Area PF Complex	R Area 31 pd
L Area 10v	L Medial Superior temporal Area	R Area 8Av^	
L Third Visual Area	R Area 10r^		
R Area 10v	R Area 8Av^		
Volume SCN			
L Area 52	L Area V3B	L Second Visual Area	R Ventral IntraParietal Complex
		L Auditory 5 Complex	R Area 31 pd
		L Frontal Opercular Area 4	R Area TG dorsal
		R Medial Belt Complex	L Middle Temporal Area
			R Para-Insular Area
Surface ARea SCN			
R Frontal Opercular Area 3	L Eigth Visual Area	L PreCuneus Visual Area	R Medial Superior temporal Area
R Area 31a	L Area 10v	R Auditory 4 Complex	L Third Visual Area
R Area IntraParietal 1	L Area 11 l	R Area 8BM	L VentroMedial Visual Area 3
L Area 31 pd	L Dorsal Transitional Visual Area	L Medial Area 7A	L Second Visual Area
L RetroInsular Cortex			L Area 1
R Area 6 anterior			R Second Visual Area
			L Area 52
			R Frontal Opercular Area 2

Regions identified as hubs in all groups in the CT, GMV, and SA SCNs. * indicates regions that show overlap between non-schizophrenia and HC, and ^indicates regions that show overlap between non-schizophrenia and FEAP.

*FEAP* first-episode antipsychotic-naïve patient, *SCN* structural covariance network, *Hub overlap between non-schizophrenia and healthy control, ^hub overlap between non-schizophrenia and FEAP.

### Comparison of FEAP, schizophrenia, and non-schizophrenia with HC

#### Morphometric comparisons

Sixty out of 358 regions (≈ 17%) were significantly different between FEAP and HC with small effect sizes (Fig. 1, Supplemental Table 2). Two regions showed differences in all three morphometric features, namely left Area PGs (located at the superior surface of the angular gyrus) and right Area TE1 Middle. Thirty-three regions for GMV, 8 for SA, and 36 for CT were significantly different between FEAP and HC (the total number of regions do not add up because of overlap of morphometric features). Eighty out of 358 regions were significantly different between schizophrenia and HC, 24 between non-schizophrenia and HC, and 46 between schizophrenia and non-schizophrenia in one or more of morphometric features. Left area lateral intraparietal dorsal showed changes in all three morphometric features for schizophrenia-HC comparison, right Frontal Eye Fields for schizophrenia-non-schizophrenia comparison but none between non-schizophrenia and HC. All group differences in the subgroup analysis were of large effect sizes (Supplemental Table 3).

#### Graph metrics

**Figure 2:**
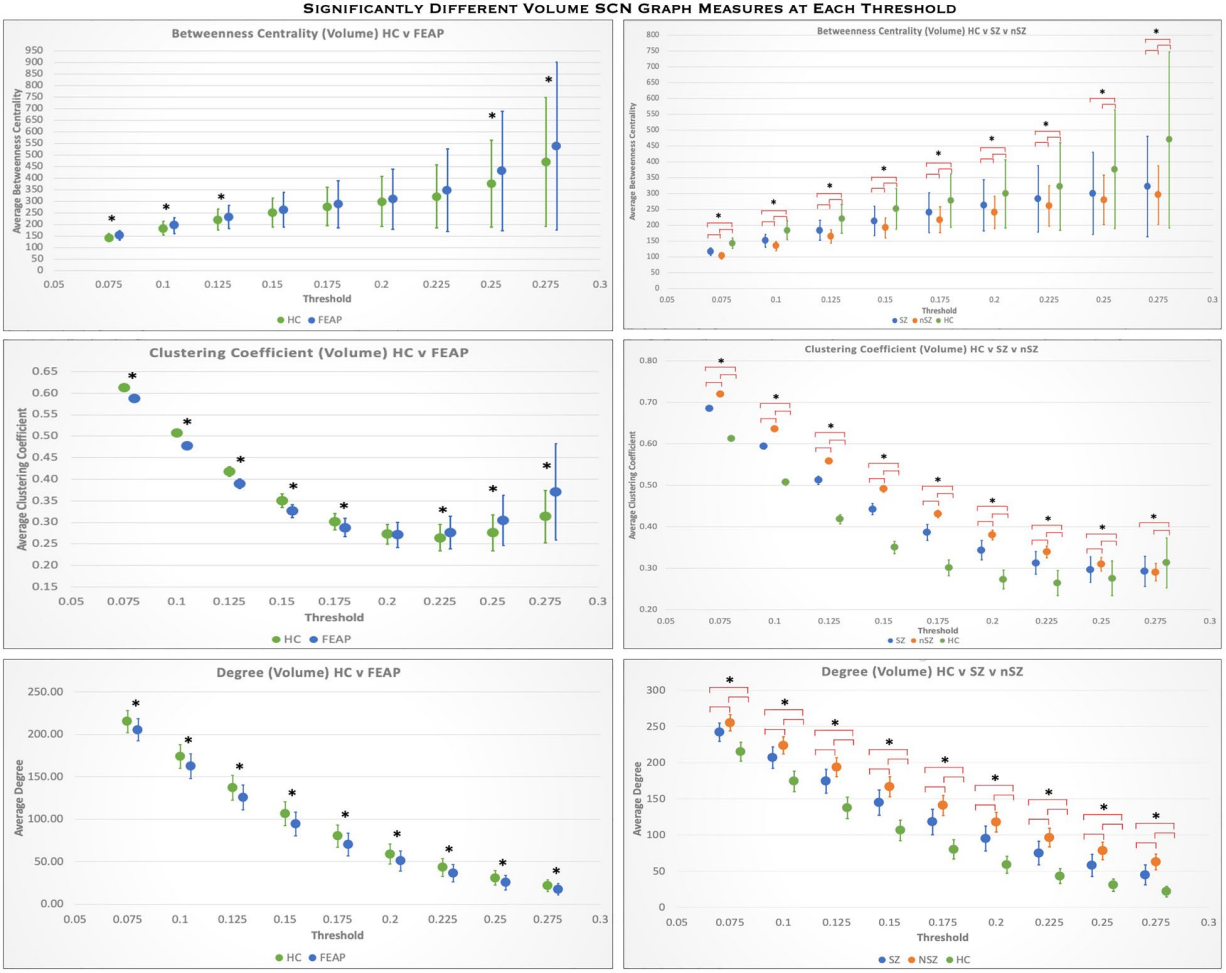
Comparison of nodal graph measures averaged across all nodes at threshold intervals of 0.025 across the small-worldness range for GMV. Top left: FEAP (blue) compared to HC (green) for betweenness centrality. Top right: Betweenness centrality across the threshold range for schizophrenia (Blue) and non-schizophrenia (Orange) compared to HC (Green). Middle left: FEAP (blue) compared to HC (green) for clustering coefficient. Middle right: Betweenness centrality across the threshold range for schizophrenia (Blue) and non-schizophrenia (Orange) compared to HC (Green). Bottom left: FEAP (blue) compared to HC (green) for degree. Bottom right: Betweenness centrality across the threshold range for schizophrenia (Blue) and non-schizophrenia (Orange) compared to HC (Green). *represents significance between groups. Error bars indicate standard deviation. See Table 1 for graph measure values averaged across the small-worldness threshold range.

**Figure 3:**
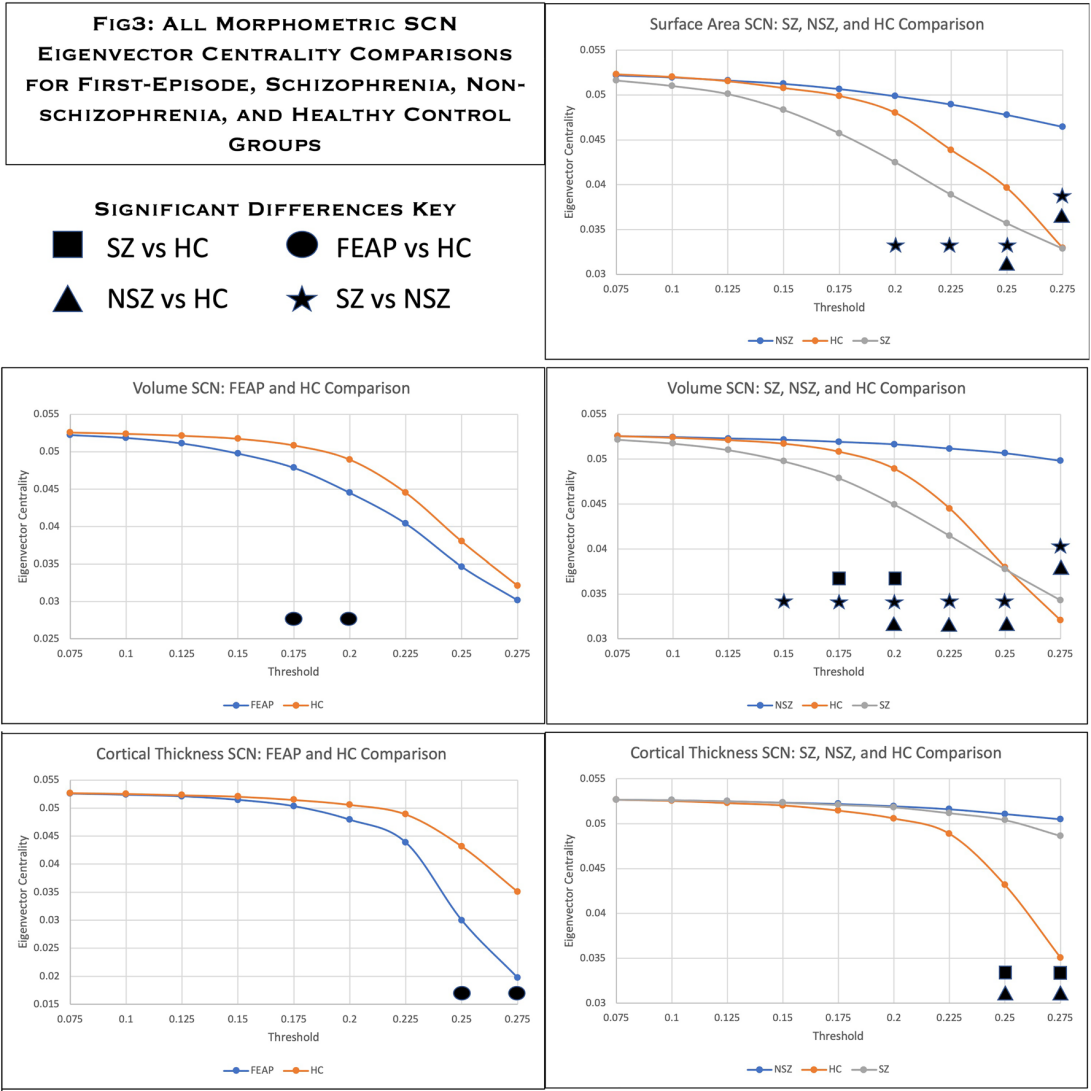
Statistically significant eigenvector centrality group comparisons from all morphometric SCNs; SA (top), volume (middle), and CT (bottom). FEAP (blue) is compared to controls (orange) on the left and schizophrenia (gray) and non-schizophrena (blue) are compared to controls (orange) on the right. Comparisons were done at each threshold across the small-worldness range. Statistically significant differences are indicated by unique symbols for each type of compasion in the key at the top left of the Figureure.

Graph metrics showed similar differences in each of the SCNs of GMV, CT and SA of FEAP compared to controls. Degree was lower and BC was higher (both nodal metrics) in FEAP compared to controls (Table 1, Supplemental Table 4). CC was significantly lower at lower thresholds and higher at higher thresholds for FEAP (Fig. 2, Supplemental Table 4). Schizophrenia and non-schizophrenia both had higher degree and lower BC compared to HC (Table 1, Fig. 2, Supplemental Table 4). Compared to non-schizophrenia, schizophrenia had lower degree, higher BC, and lower CC. EC did not follow a pattern like the other nodal graph measures but did show significant differences between groups (Table 1, Fig. 3). The global measures were not significantly different between groups after multiple comparison corrections. All the regions that showed significant morphometric differences between groups did not show significant differences in CC, BC, or degree after multiple comparison corrections using Bonferroni approach.

#### Modular structure and hubs

**Figure 4:**
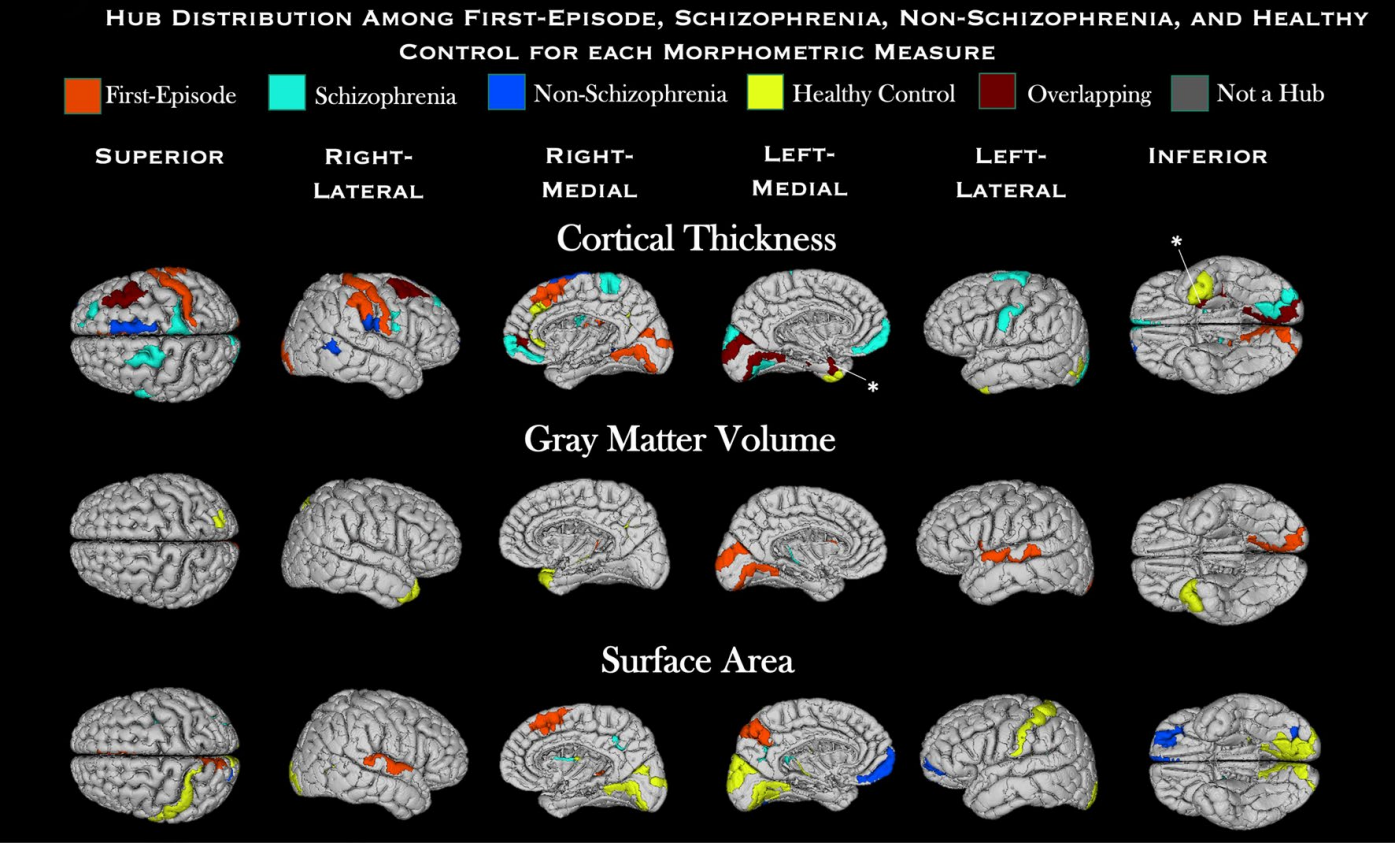
*Hub Distribution.* Hubs are shown in red for FEAP, yellow for HC, light blue for schizophrenia, and dark blue for non-schizophrenia for cortical thickness (top), gray matter volume (middle), and surface area (bottom) SCNs. Gray areas are not defined as hubs for any group and dark red areas are overlapping between groups. *signifies the hubs overlapping between HC and NSZ and all other hubs that overlap are between FEAP and NSZ. For names of hub regions see Table 2.

The number of modules increased with the threshold across the σ range (Supplemental Table 5). The modules consisted of common and unique regions among the groups. The FEAP modules consisted of relatively fewer prefrontal, medial and lateral temporal, auditory, visual, and parietal regions compared to HC. Average nodal degree and density within the modules did not differ between FEAP and HC. Hubs were distributed across 3 communities in FEAP and 2 in HC for GMV, 3 in FEAP and 4 in HC for SA, and 2 in FEAP and 4 in HC for CT. Although there were equal number of hubs in FEAP and HC, they were unique to each group in the GMV, CT, and SA networks. FEAP hubs were located in prefrontal, visual, and auditory areas whereas the HC hubs were more widespread in the cingulate, visual, temporal, and prefrontal regions (Fig. 4, Table 2). The number of hubs in the SCN of schizophrenia and non-schizophrenia were similar for the SCNs of GMV, SA and CT (Table 2). Three hub regions overlapped between the non-schizophrenia and FEAP groups and one hub overlapped between non-schizophrenia and HC groups in the cortical thickness SCN only but hubs in SZ SCN did not overlap with HC or NSZ SCNs (Table 2).

#### Network resilience

**Figure 5:**
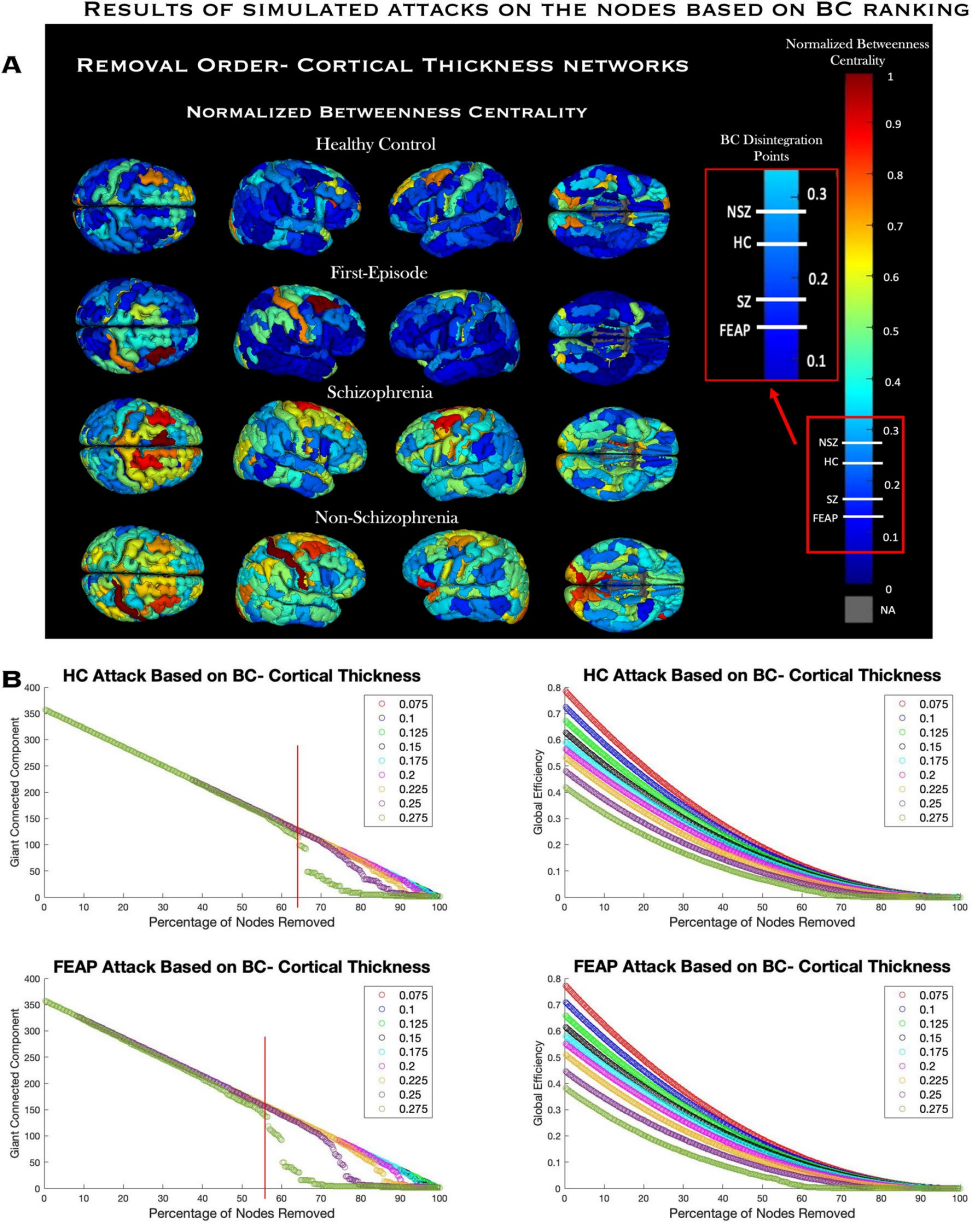

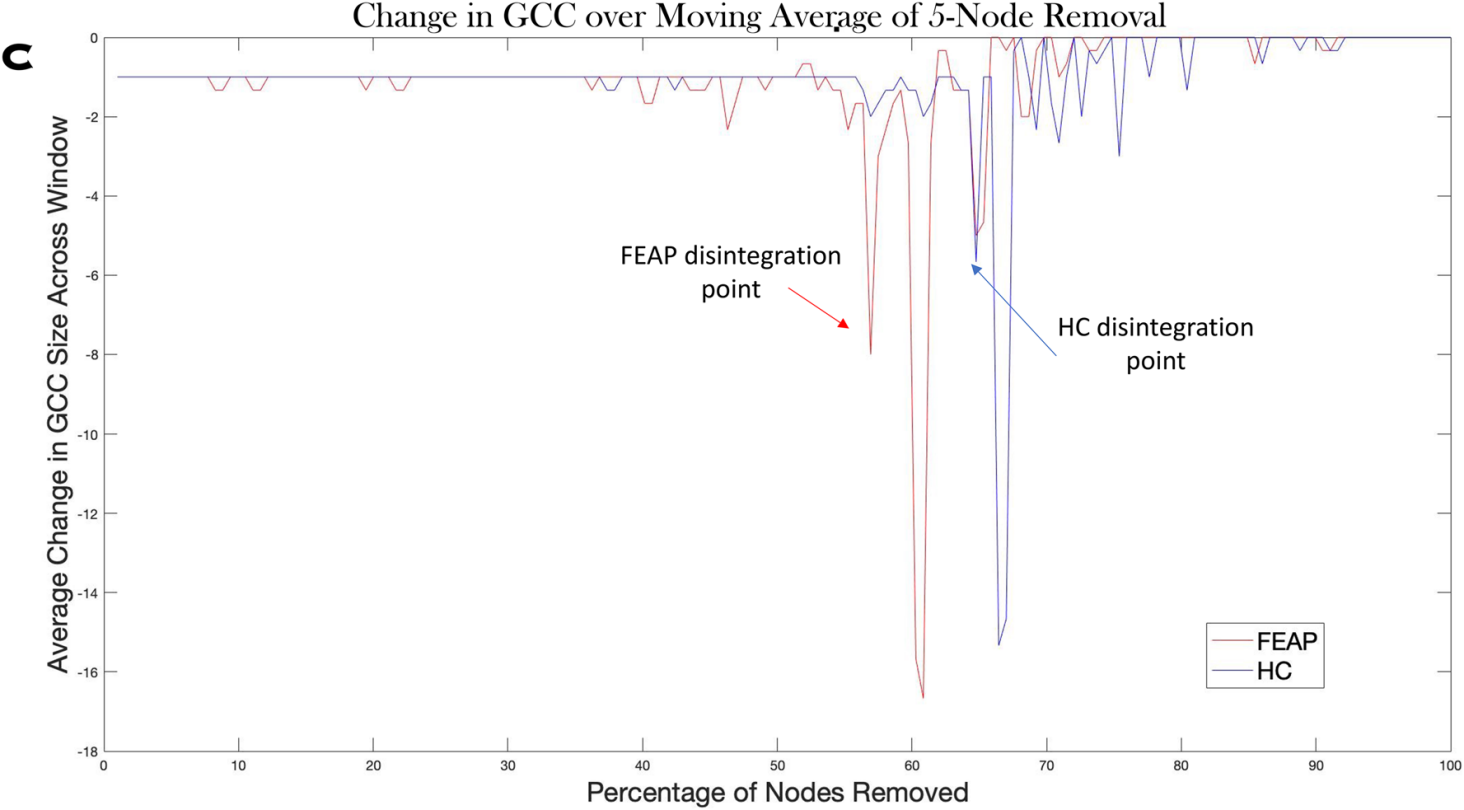
Results of simulated attacks on the nodes based on BC ranking. Top Panel A: Order of nodes “attacked” based on betweenness centrality: Normalized betweenness centrality distribution throughout all brain regions for all groups in the cortical thickness SCN. A region colored in dark red represents the highest BC and was removed first in the cumulative sequential attack simulations, and dark blue represents the lowest BC and was removed last. The normalized BC for each group at the first point of disintegration is shown in the magnified color bar. Gray regions, NA on the color bar, are subcortical nodes that were not in the analysis (see supplemental material Fig. 1 for volume and surface area BC and EC projections). All Figureures in panel A are at a threshold of 0.275, the threshold where the simulations were most impactful. Middle panel B: GCC (middle left) and global efficiency (middle right) for HC and FEAP during the cumulative sequenctial attacks, A: shows the network “attack” based on BC using CT SCNs for both FEAP (middle) and HC (top) across the small-worldness range. Red line indicates first disintegration point. Bottom Figureure C : Change in GCC . The Figureure shows the change in GCC size during the removals using a moving average window. Arrows indicate for each group where the change in GCC was three times the window prior.

FEAP GCC size started becoming unstable (reduction in GCC by > 1 node but < 5 nodes moving average with the removal of one node in the previous step) following removal of 8% of nodes with the highest BC whereas HC needed ≈36% node removal in the sequential cumulative “attack”. Nodes were removed in order of highest to lowest BC, and FEAP SCN disintegrated when a node with ~ 14% of the max BC measure was removed whereas schizophrenia, HC, and non-schizophrenia nodes were 18%, 24%, and 28% of the max BC measure respectively (Fig. 5A, Supplemental Fig. 1). Alternatively, FEAP SCN showed disintegration when 56.4% of the nodes were “attacked” in the descending order of BC while HC SCN needed 64.2% nodes “attacked” at σ threshold of 0.275 (Fig. 5B,C). Schizophrenia SCN disintegrated with 78% of nodes removed and non-schizophrenia at 85% compared to their age/sex matched HC groups at 81% and 88%, respectively (Supplemental Fig. 2B). At lower thresholds within the σ-range, more nodes needed removal for disintegration in all groups with no differences when the threshold was 0.175 or lower. Global efficiency did not show group-differences at any threshold (Fig. 5; Supplemental Fig. 2). There were no differences in GCC or global efficiency at any threshold with either of the non-cumulative “attacks.”

**Figure 6:**
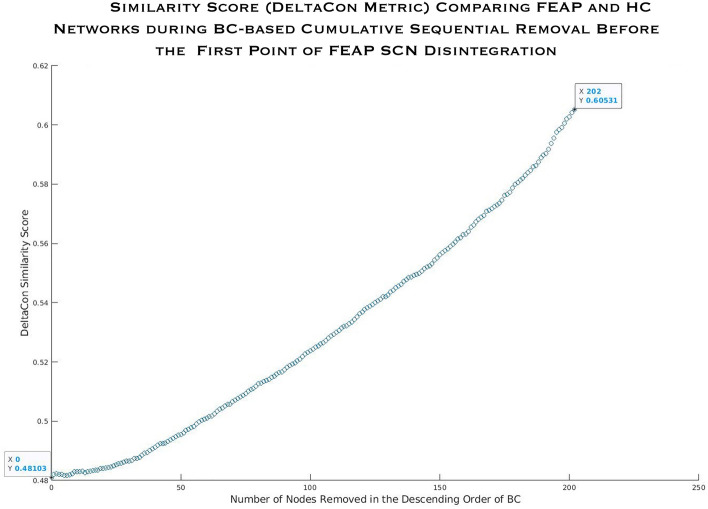
Similarity score before and after each sequential node removal during the targeted attack on betweenness centrality until FEAP disintegration point. Nodes were removed in descending order of betweenness centrality. Boxes highlight the similarity score before removals (0.48) and after 202 nodes were removed (0.61).

The *DeltaCon* similarity metric before the sequential cumulative attack was 0.481 and after removing 56.4% (n = 202) of nodes with highest BC or EC (where FEAP SCN started to disintegrate) in both FEAP and HC SCN, it was 0.605 (Fig. 6). Of the 202 nodes removed in both FEAP and HC, 126 were common between the groups consisting of prefrontal (n = 34), insula/orbitofrontal (n = 20), parietal (n = 16), sensorimotor (n = 15), visual (n = 14), and others. In the 77 unique nodes removed in each group, prefrontal, parietal, and visual nodes constituted about 2/3rds among FEAP whereas in HC SCN, these nodes were in the prefrontal, parietal, visual, insula/orbitofrontal area, and auditory areas. For schizophrenia and non-schizophrenia compared to HC, the DeltaCon similarity metric before the attack was 0.456 and 0.459 and after removing nodes until disintegration the score was 0.594 and 0.419, respectively.

### Association with psychopathology

SCNs of FEAP scoring above and below the median SANS and SAPS scores showed significantly higher degree and CC, but lower BC compared to full HC SCN (Bonferroni corrected *p* < 0.05). However, SCN for FEAP scoring above the SANS and SAPS median score had significantly higher degree and CC, but lower BC compared to SCN of FEAP scoring below SANS and SAPS median score (Bonferroni corrected *p* < 0.05). Greater severity of positive and negative symptoms was associated with higher degree and CC, and lower BC. In addition, greater negative symptom severity was associated with twice the magnitude of changes in the SCN metrics which was statistically significant. All differences were of small/medium effect size.

## Discussion

Major findings of our study are that the nodal but not global graph metrics showed significant differences in patient groups compared to HC, all three morphometric SCNs showed similar case-control differences in graph metrics, FEAP SCN was more vulnerable to sequential cumulative attacks compared to controls, and negative symptom severity was associated with greater magnitude of network disarray. In addition, although the number of modules did not significantly differ between the groups, nodal composition of modules of patients were different compared to that of controls. There were equal number of hubs in patients compared to controls, but these were more spatially distributed in controls. While severity of negative and positive symptoms was associated with lower BC, higher CC, and higher degree, negative symptoms were associated with more drastic changes in graph properties compared to positive symptom severity. To our knowledge, this is the first study to use the connectivity-based multi-modal Glasser parcellation based on HCP atlas to comprehensively examine high-resolution SCNs of three morphometric features, namely GMV, CT, and SA, among FEAP patients using a graph theoretic approach, and comprehensively examine network resilience.

The HCP-based atlas provides better insight on the connectivity of SCNs because the regions-of-interests are structurally and functionally connected. For example, Area Intraparietal 1, a schizophrenia hub with lower SA and GMV among schizophrenia patients compared to controls, is located in the inferior parietal cortex and associated with processing language, math, motor cue, and faces-shapes functionally contrasts more than other neighboring areas^36^. It is functionally connected with area 6a in premotor region, another schizophrenia hub, through the superior longitudinal fasciculus^37^, which showed differences in diffusion properties in diffusion MRI data previously^38,39^. A HC hub, anterior 24, which is part of the cingulate cortex is functionally connected to regions in the medial and lateral frontal lobe, temporal lobe, lateral parietal, and posterior cingulate with white matter fibers in the cingulum ^40^. Future research should investigate the validity of such HCP atlas SCN-based observations using functional MRI data.

FEAP subjects showed no differences in global graph measures which suggests that local and nodal network differences may be pathophysiologically more important. Fewer edges per node on an average (degree), and a higher frequency of connecting with neighboring nodes than distant nodes (CC) at higher thresholds, and more nodes in the shortest path between other nodes (BC) in FEAP SCN compared to controls suggests that the nodal connectivity was not as uniform in the FEAP network as the HC SCN. The FEAP network appeared to be relatively sparsely connected but is interspersed with highly connected nodes with neighboring nodes with a higher centrality of nodes compared to HC SCN. Hubs were more uniformly distributed across the brain in HC compared to FEAP hubs that were concentrated in a few regions. Since hubs facilitate transfer of information across the network, such network architecture may result in inefficient network communication that could contribute to clinical symptoms of psychotic disorders^41^ Additionally, overlap of hubs of non-schizophrenia SCN with FEAP and HC groups suggests that non-schizophrenia network connectivity may have similarities with HC and FEAP. Non-overlap of hubs of schizophrenia with other groups suggests that the communication pattern within the schizophrenia network may be unique. Since we are the first to report this finding, independent replications may identify targets of treatment development, especially with the emerging electroceutical approaches.

To further support the above possibility, modular structure, an “optimized” network organization^42^ for communication across spatially separated regions, was different between SCNs of FEAP and HC possibly representing different connectional patterns among different regions in separate modules. Further, similar average nodal degree within the modules in FEAP and SCN in the background of lower degree in FEAP SCN suggests that the FEAP SCN is even less sparsely connected outside the modules. These modular properties together with perturbed network architecture among FEAP could substantially affect neurobiological processes underlying psychopathology.

Since GMV is a product of SA and CT which are differentially affected by the disease and developmental processes, finding differences in each of these measures could help determine the pathophysiology. For example, CT is related to laminar architecture, highly conserved phylogenetically^43^, and related to altered neuropil density, neuronal packing density, soma volumes, and myelination^44^⁠. Growth in SA is closely tied to cortical curvature and may be determined by the number of cortical columns^45^. Region-specific cortical thinning has been observed in schizophrenia^46,47^ and in familial high-risk subjects who converted to psychosis^48,49^. However, we found that the network properties were similar between the morphometric features suggesting that the putative neurobiological processes regulating CT, SA and GMV did not have major impact on the covariance structure. This was further supported by the comparison of graph metrics for regional segregation and centrality for regions showing group differences in morphometrics. None of the regions in any SCN for any comparison showed significant differences in degree, CC, or BC suggesting that morphometrically different regions may not differ in network connectivity when observed in isolation. Instead, the overall covariance structure among all the nodes is important and observed changes in network connectivity arise from the combined influences of these morphometrically different regions on the covariance structure of the network.

Our observations suggests that the FEAP network would be less resilient than HC SCN. Sequential cumulative BC attacks had the largest effect on network integrity suggesting that nodes with high centrality may be vital for network integrity. We predicted that the global efficiency would decrease because removing nodes with highest BC would increase the pathlength, but it did not, suggesting that network disintegration may not alter some network features. Further, non-significant change in resilience with non-cumulative attacks suggests that no single node has paramount importance on the SCN integrity. Previous studies analyzing schizophrenia with the AAL atlas showed reductions in GCC along with global efficiency after cumulative removals^18^. Our findings suggest that in the early course of the disease, the global properties such as efficiency may be preserved even during network disintegration whereas the chronic schizophrenia network did not. This is supported by a resting fMRI network showing no loss of resilience in childhood onset schizophrenia^19^.

Our hypothesis on the similarity score becoming higher as the top centrality nodes are removed was supported by our data. Among the nodes removed, 62% were common between the FEAP and HC SCNs. Among the nodes unique to each group, FEAP nodes were mainly concentrated in the prefrontal, parietal and visual areas that might have made the network less similar whereas HC nodes were more widely distributed across different areas suggesting that such wider distribution of nodes may underlie higher resilience of the HC network. In addition, wider distribution of hubs and modular components in HC may also contribute. Further, increasing similarity score with sequential cumulative BC/EC-based attacks suggests that these nodes distinguish the SCNs and may be pathophysiologically significant.

Association of graph metrics with severity of psychopathology showed that associations were quantitative in the same direction but not qualitative. Greater severity of both positive and negative symptoms was associated with higher degree and CC and lower BC, but the association of the severity of negative symptoms with twice the magnitude of changes in the SCN metrics suggests that greater network disarray may underlie severity of negative symptoms. This observation may support the relative resistance of negative symptoms to treatment and possibly the association with poorer outcome.

Schizophrenia and non-schizophrenia groups showed significant differences in BC, CC, and degree but in opposite directions than the FEAP SCN compared to the HC. Although relatively small sample size might have contributed, other possibilities should be considered. In this sample, non-schizophrenia SCN was more similar to HC than schizophrenia or FEAP. Therefore, it is likely that the SCN in more severe psychotic disorders may comprise of nodes of lower degree and higher BC compared to HC and relatively less severe disorder group of non-schizophrenia. To partly address potential contributions of sample size to the results, we conducted bootstrapping to ensure stable measures from random networks were used to build the threshold range and compare the patient groups. Because of the unequal sample sizes, we did not break down our FEAP sample into subgroups of schizophrenia spectrum disorders (n = 62) and affective psychosis (n = 17).

There are several strengths in our study. We have examined FEAP which minimizes the impact of illness duration and medications to better estimate disease effects on SCNs. This is the first study to examine high-resolution SCN of multiple morphometric features. We have used appropriate corrections for multiple tests throughout the study, controlling for age, sex, and total brain morphometrics using partial correlations, and examination of graph metrics over σ range where each network is non-random and remains fully connected. We excluded weakly correlated nodes to reduce the noise in the network attempting to represent the network in a biologically relevant manner^50^. Examination of resilience more comprehensively highlighted distinct qualitative differences between the networks. Limitations include a modest sample size. Although we used 1.5 T imaging data, the resolution was adequate to implement reliable parcellation and morphometry. Even though 3 and 7 Tesla scanners may enhance the accuracy of segmentation and parcellations, this advantage alone is unlikely to affect the results since we have treated all study groups alike with the same approach for quality check for noise and for accuracy of parcellation that were confirmed by visual inspection, as well. While the cross-sectional nature of the study is a limitation, these studies are important to investigate unexplored areas of morphometric variations and avoid challenges of longitudinal studies on this population such as medication effects. Precise neurobiological impact is difficult to quantify in SCNs that are averaged across the groups. Emerging methods, e.g., structural similarity network (SSN)^49^ and individualized differential SCN^51^ analyses produce morphological networks for each subject that allow one to correlate with clinical measures using graph metrics.

## Supplementary Information

Supplementary Information.

## Data Availability

The datasets analyzed in the current study are not publicly available because it was not routine for participants to be asked for their consent to share data publicly when this data was collected, and therefore, subjects did not consent. The parent dataset is not under the authors’ sole control. Reasonable requests to access the dataset for scientific purposes should be addressed to the corresponding author by qualified investigators to consider sharing the data.

## References

[1] Shenton ME, Dickey CD, Frumin M, McCarley RW (2001). A review of MRI findings in schizophrenia. Schizophr. Res..

[2] Wright IC (2000). Meta-analysis of regional brain volumes in schizophrenia. Am. J. Psychiatry.

[3] Kennedy DN (1998). Gyri of the human neocortex: An MRI-based analysis of volume and variance. Cereb. Cortex.

[4] Lerch JP (2006). Mapping anatomical correlations across cerebral cortex (MACACC) using cortical thickness from MRI. Neuroimage.

[5] Alexander-Bloch A, Giedd JN, Bullmore E (2013). Imaging structural co-variance between human brain regions. Nat. Rev. Neurosci..

[6] Gong G, He Y, Chen ZJ, Evans AC (2012). Convergence and divergence of thickness correlations with diffusion connections across the human cerebral cortex. Neuroimage.

[7] Geerligs L, Cam C, Henson RN (2016). Functional connectivity and structural covariance between regions of interest can be measured more accurately using multivariate distance correlation. Neuroimage.

[8] Mechelli A, Friston KJ, Frackowiak RS, Price CJ (2005). Structural covariance in the human cortex. J. Neurosci. Off. J. Soc. Neurosci..

[9] Griffa A (2015). Characterizing the connectome in schizophrenia with diffusion spectrum imaging. Hum. Brain Mapp..

[10] Shi F (2012). Altered structural connectivity in neonates at genetic risk for schizophrenia: A combined study using morphological and white matter networks. Neuroimage.

[11] Cauda F (2018). The morphometric co-atrophy networking of schizophrenia, autistic and obsessive spectrum disorders. Hum. Brain Mapp..

[12] Xu L, Pearlson G, Calhoun VD (2009). Joint source based morphometry identifies linked gray and white matter group differences. Neuroimage.

[13] Zugman A (2015). Structural covariance in schizophrenia and first-episode psychosis: An approach based on graph analysis. J. Psychiatr. Res..

[14] Nelson EA, White DM, Kraguljac NV, Lahti AC (2018). Gyrification connectomes in unmedicated patients with schizophrenia and following a short course of antipsychotic drug treatment. Front. Psychiatry.

[15] Wannan CMJ (2019). Evidence for network-based cortical thickness reductions in schizophrenia. Am. J. Psychiatry.

[16] Prasad K (2022). Structural covariance networks in schizophrenia: A systematic review Part II. Schizophr. Res..

[17] Prasad K (2022). Structural covariance networks in schizophrenia: A systematic review Part I. Schizophr. Res..

[18] Palaniyappan L (2019). Structural covariance and cortical reorganisation in schizophrenia: A MRI-based morphometric study. Psychol. Med..

[19] de Arruda GF, Fontoura Costa L, Schubert D, Rodrigues FA (2014). Structure and dynamics of functional networks in child-onset schizophrenia. Clin. Neurophysiol. Off. J. Int. Fed. Clin. Neurophysiol..

[20] Koutra D, Shah N, Vogelstein JT, Gallagher B, Faloutsos C (2016). DELTACON: Principled massive-graph similarity function with attribution. ACM Trans. Knowl. Discov. Data.

[21] Glasser MF (2016). A multi-modal parcellation of human cerebral cortex. Nature.

[22] Mitelman SA, Shihabuddin L, Brickman AM, Buchsbaum MS (2005). Cortical intercorrelations of temporal area volumes in schizophrenia. Schizophr. Res..

[23] Mitelman SA, Byne W, Kemether EM, Hazlett EA, Buchsbaum MS (2006). Correlations between volumes of the pulvinar, centromedian, and mediodorsal nuclei and cortical Brodmann’s areas in schizophrenia. Neurosci. Lett..

[24] Alexander-Bloch A (2012). The discovery of population differences in network community structure: New methods and applications to brain functional networks in schizophrenia. Neuroimage.

[25] Bassett DS (2008). Hierarchical organization of human cortical networks in health and schizophrenia. J. Neurosci. Off. J. Soc. Neurosci..

[26] Zhang Y (2012). Abnormal topological organization of structural brain networks in schizophrenia. Schizophr. Res..

[27] Prasad KM, Patel AR, Muddasani S, Sweeney J, Keshavan MS (2004). The entorhinal cortex in first-episode psychotic disorders: A structural magnetic resonance imaging study. Am. J. Psychiatry.

[28] Andreasen NC (1984). Scale for the Assessment of the Positive Symptoms (SAPS).

[29] Andreasen NC (1984). Scale for the Assessment of Negative Symptoms (SANS).

[30] Mills, K. (ed figshare) (2016).

[31] Neurolab, C. HCP-MMP1.0 volumetric (NIfTI) masks in native structural space (2017).

[32] Maslov S, Sneppen K (2002). Specificity and stability in topology of protein networks. Science.

[33] Rubinov M, Sporns O (2010). Complex network measures of brain connectivity: Uses and interpretations. Neuroimage.

[34] Blondel VD, Guillaume J-L, Lambiotte R, Lefebvre E (2008). Fast unfolding of communities in large networks. J. Stat. Mech. Theory Exp..

[35] Tantardini M, Ieva F, Tajoli L, Piccardi C (2019). Comparing methods for comparing networks. Sci. Rep..

[36] Baker CM (2018). A connectomic atlas of the human cerebrum–Chapter 6: The temporal lobe. Oper. Neurosurg..

[37] Baker CM (2018). A connectomic atlas of the human cerebrum-Chapter 7: The lateral parietal lobe. Oper. Neurosurg. (Hagerstown).

[38] Prasad KM, Upton CH, Schirda CS, Nimgaonkar VL, Keshavan MS (2015). White matter diffusivity and microarchitecture among schizophrenia subjects and first-degree relatives. Schizophr. Res..

[39] Ellison-Wright I, Bullmore E (2009). Meta-analysis of diffusion tensor imaging studies in schizophrenia. Schizophr. Res..

[40] Baker CM (2018). A connectomic atlas of the human cerebrum-Chapter 4: The medial frontal lobe, anterior cingulate gyrus, and orbitofrontal cortex. Oper. Neurosurg. (Hagerstown).

[41] van den Heuvel MP, Fornito A (2014). Brain networks in schizophrenia. Neuropsychol. Rev..

[42] Sporns O, Betzel RF (2016). Modular brain networks. Annu. Rev. Psychol..

[43] Rakic P (2009). Evolution of the neocortex: A perspective from developmental biology. Nat. Rev. Neurosci..

[44] Deoni SC (2011). Mapping infant brain myelination with magnetic resonance imaging. J. Neurosci. Off. J. Soc. Neurosci..

[45] Rakic P (1988). Specification of cerebral cortical areas. Science.

[46] Schultz CC (2010). Reduced cortical thickness in first episode schizophrenia. Schizophr. Res..

[47] Narr KL (2005). Mapping cortical thickness and gray matter concentration in first episode schizophrenia. Cereb. Cortex.

[48] Bois C (2015). Cortical surface area differentiates familial high risk individuals who go on to develop schizophrenia. Biol. Psychiatry.

[49] Homan P (2019). Structural similarity networks predict clinical outcome in early-phase psychosis. Neuropsychopharmacol. Off. Publ. Am. Coll. Neuropsychopharmacol..

[50] Bassett DS, Bullmore E (2006). Small-world brain networks. Neuroscientist.

[51] Liu Z (2021). Resolving heterogeneity in schizophrenia through a novel systems approach to brain structure: Individualized structural covariance network analysis. Mol. Psychiatry.

