# Supplementary material for: Modular architecture and resilience of structural covariance networks in first-episode antipsychotic-naive psychoses
Source: Sci Rep. 2023 May 12;13:7751. doi: 10.1038/s41598-023-34210-y (PMC10181992; doi:10.1038/s41598-023-34210-y)
Supplement: Supplementary file 1 — Supplementary Information. [file 41598_2023_34210_MOESM1_ESM.docx]

Supplemental Table 1: Introduction to graph metrics used in the analysis. All definitions are from the Brain Connectivity Toolbox or Prasad et al (https://doi.org/10.1016/j.schres.2021.11.036).

| **Global and Nodal Graph Measures** | | |
| --- | --- | --- |
|  | **Type of Measure** | **Definition** |
| **Global Measures** | | |
| **Characteristic Pathlengh** | Integration | Average of the number of edges appearing in the shortest paths linking pairs of nodes in the graph, where “shortest” refers to the fewest edges traversed not to a physical distance between nodes. |
| **Assortativity** | Segregation | The correlation coefficient of degree between pairs of connected nodes in a network. |
| **Modularity** | Segregation | Modularity measures the fraction of edges that connect nodes in the same community, relative to the same fraction produced under random conditions. |
| **Efficiency** | Integration | The reciprocal of the characteristic path length. Networks with higher efficiency have lower average pathlength. |
| **Nodal Measures** | | |
| **Degree** | Centrality | Number of edges a node is associated with. |
| **Clustering Coefficient** | Segregation | CC quantifies the degree to which connected nodes share neighbors. |
| **Betweenness Centrality** | Centrality | A number between 0 and 1 corresponding to the fraction of shortest paths between pairs of nodes that pass through a node. |
| **Eigenvector Centrality** | Centrality | A measure of the influence of a node on the network computed by determining the extent to which a node connects to high degree nodes. |
| **Eccentricity** | Integration | Eccentricity is the maximal shortest path length between a node and any other node |

Supplemental Table 2: Demographics for all groups used in study. For age, mean and standard deviation is shown.

| **Group** | **Age** | **Sex** |
| --- | --- | --- |
| First-Episode Anti-psychotic Naïve (n=79) | 23.99 ± 7.22 years | 56 Males |
| Healthy Control (n=68) | 24.59 ± 6.63 years | 39 Males |
| Schizophrenia (n=46)* | 24.80 ± 6.26 years | 33 Males |
| Healthy Control (Age and sex matched for Schizophrenia) (n=43) | 24.60 ± 6.69 years | 23 Males |
| Non-Schizophrenia (n=33)^#^ | 22.87 ± 8.35 years | 23 Males |
| Healthy Control- (Age and sex matched for Non-Schizophrenia) (n=34) | 23.13 ± 7.76 years | 22 Males |

* Includes Schizophrenia (n=41) and Schizoaffective disorder (n=5)

^#^ Includes Delusional disorder (n=2), psychotic disorder not otherwise specified (n=12), bipolar disorders (n=4), depressive disorders (n=13), unspecified mood disorders (n=1), and schizophreniform disorder (n=1)

Supplemental Table 3: Morphometric differences in volume, cortical thickness and surface area between FEAP, SZ, NSZ, and controls of the regions that showed differences in at least two of the morphometric measure.

| **Morphometric Comparisons- FEAP vs HC** | | | | | | | | | | | | | | | | | | |
| --- | --- | --- | --- | --- | --- | --- | --- | --- | --- | --- | --- | --- | --- | --- | --- | --- | --- | --- |
|  | Cortical Thickness | | | | | | Volume | | | | | | Surface Area | | | | | |
| Region Name | FEAP Mean ± SD (mm) | HC Mean ± SD (mm) | Difference | Cohen’s D | F | Sig. | FEAP Mean ± SD (mm^3^) | HC Mean ± SD (mm^3^) | Difference | Cohen’s D | F | Sig. | FEAP Mean ± SD (mm^2^) | HC Mean ± SD (mm^2^) | Difference | Cohen’s D | F | Sig. |
|  | Cortical Thickness, Volume, and Surface Area | | | | | | | | | | | | | | | | | |
| L Area PGs | 2.62±0.21 | 2.68±0.17 | -2.28% | -0.33 | 4.57 | 0.03 | 2951.58±522.20 | 3215.93±535.80 | -8.22% | -0.50 | 14.59 | <.001 | 943.13±160.81 | 994.78±154.31 | -5.19% | -0.33 | 6.70 | 0.01 |
| R Area TE1 Mid | 2.61±0.32 | 2.71±0.26 | -3.63% | -0.34 | 4.59 | 0.03 | 1507.3±386.79 | 1669.68±345.16 | -9.72% | -0.44 | 12.94 | <.001 | 475.16±89.13 | 498.01±80.94 | -4.59% | -0.27 | 6.05 | 0.02 |
|  | Volume and Surface Area | | | | | | | | | | | | | | | | | |
| L Sup 6-8 Trans A |  |  |  |  |  |  | 801.62±232.34 | 881.13±225.54 | -9.02% | -0.35 | 7.99 | 0.01 | 242.49±51.40 | 252.46±41.75 | -3.95% | -0.21 | 4.10 | 0.05 |
| R A TE1 post |  |  |  |  |  |  | 2910.16±594.02 | 3085.38±562.04 | -5.68% | -0.30 | 8.30 | 0.01 | 875.67±159.26 | 911.49±144.90 | -3.93% | -0.24 | 4.23 | 0.04 |
| R Sixth Visual A |  |  |  |  |  |  | 672.89±165.24 | 740.63±178.44 | -9.14% | -0.39 | 7.99 | 0.01 | 365.76±74.25 | 385.87±74.25 | -5.21% | -0.27 | 4.05 | 0.05 |
|  | Cortical Thickness and Volume | | | | | | | | | | | | | | | | | |
| L A 3a | 1.87±0.11 | 1.91±0.10 | -1.77% | -0.32 | 6.45 | 0.01 | 1147.54±133.58 | 1189.24±125.36 | -3.51% | -0.32 | 6.05 | 0.02 |  |  |  |  |  |  |
| L A 47m | 2.64±0.32 | 2.73±0.35 | -3.40% | -0.28 | 4.11 | 0.04 | 409.22±102.11 | 427.59±86.22 | -4.30% | -0.19 | 4.29 | 0.04 |  |  |  |  |  |  |
| L Ventral A 6 | 3.07±0.29 | 3.17±0.30 | -2.96% | -0.32 | 4.64 | 0.03 | 991.78±216.26 | 1031.9±208.30 | -3.89% | -0.19 | 3.98 | 0.05 |  |  |  |  |  |  |
| L A Ant 32 prime | 2.74±0.19 | 2.84±0.19 | -3.44% | -0.52 | 13.75 | <.001 | 895.63±256.05 | 976.47±237.16 | -8.28% | -0.33 | 7.36 | 0.01 |  |  |  |  |  |  |
| L Inf 6-8 Trans A | 2.61±0.22 | 2.71±0.24 | -3.67% | -0.44 | 9.08 | 0.01 | 1078.41±252.64 | 1162.15±321.97 | -7.21% | -0.29 | 5.26 | 0.02 |  |  |  |  |  |  |
| L A TE1 post | 2.68±0.22 | 2.75±0.21 | -2.54% | -0.33 | 5.82 | 0.01 | 3427.46±618.30 | 3575.09±570.00 | -4.13% | -0.25 | 6.14 | 0.01 |  |  |  |  |  |  |
| R A OP2-3/VS | 2.72±0.23 | 2.79±0.25 | -2.34% | -0.27 | 4.17 | 0.04 | 428.49±58.04 | 443.29±77.15 | -3.33% | -0.22 | 4.60 | 0.03 |  |  |  |  |  |  |
| R Suppl & Cing Eye Field | 2.93±0.29 | 3.05±0.23 | -3.70% | -0.44 | 8.80 | 0.01 | 1533.77±274.76 | 1587.84±260.28 | -3.41% | -0.20 | 5.45 | 0.02 |  |  |  |  |  |  |

| **Morphometric Comparisons- SZ vs HC** | | | | | | | | | | | | | | | | | | |
| --- | --- | --- | --- | --- | --- | --- | --- | --- | --- | --- | --- | --- | --- | --- | --- | --- | --- | --- |
|  | Cortical Thickness | | | | | | Volume | | | | | | Surface Area | | | | | |
| Region Name | SZ Mean ± SD (mm) | HC Mean ± SD (mm) | Difference | Cohen’s D | F | Sig. | SZ Mean ± SD (mm^3^) | HC Mean ± SD (mm^3^) | Difference | Cohen’s D | F | Sig. | SZ Mean ± SD (mm^2^) | HC Mean ± SD (mm^2^) | Difference | Cohen’s D | F | Sig. |
|  | Cortical Thickness, Volume, and Surface Area | | | | | | | | | | | | | | | | | |
| L A Lat Intra-Parietal Dor | 2.14±0.027 | 2.21±0.022 | -3.21% | -0.82 | 4.20 | 0.04 | 552.5±23.12 | 613.7±18.98 | -10.50% | -0.82 | 4.14 | 0.04 | 263.31±8.08 | 285.18±6.63 | -7.97% | -0.81 | 4.34 | 0.04 |
|  | Volume and Surface Area | | | | | | | | | | | | | | | | | |
| L A Intra- Parietal 1 |  |  |  |  |  |  | 1273.3±52.82 | 1428.9±43.35 | -11.52% | -0.85 | 5.13 | 0.03 | 502.91±12.19 | 543.83±10.82 | -7.82% | -0.87 | 5.70 | 0.02 |
| R A post 24 |  |  |  |  |  |  | 894.7±34.23 | 991.1±28.10 | -10.22% | -0.84 | 4.68 | 0.03 | 214.01±5.92 | 232.32±4.86 | -8.20% | -0.86 | 5.65 | 0.02 |
| R A TE2 post |  |  |  |  |  |  | 1628.3±45.48 | 1761.1±37.32 | -7.84% | -0.85 | 5.04 | 0.03 | 669.48±12.93 | 704.15±10.61 | -5.05% | -0.82 | 4.25 | 0.04 |
|  | Cortical Thickness and Volume | | | | | | | | | | | | | | | | | |
| L A 5m ventral | 2.22±0.022 | 2.32±0.018 | -4.41% | -0.93 | 12.4 | 0.001 | 699.6±14.97 | 743.0±12.28 | -6.02% | -0.85 | 4.96 | 0.03 |  |  |  |  |  |  |
| L Insular Granular | 2.77±0.027 | 2.85±0.022 | -2.85% | -0.85 | 4.99 | 0.03 | 554.9±9.69 | 581.3±7.95 | -4.65% | -0.83 | 4.38 | 0.04 |  |  |  |  |  |  |
| R A 6mp | 2.50±0.031 | 2.59±0.036 | -3.54% | -0.80 | 5.09 | 0.03 | 1763.3±45.15 | 1887.5±37.06 | -6.80% | -0.83 | 4.47 | 0.04 |  |  |  |  |  |  |
| R Frontal Eye Field | 2.38±0.036 | 2.48± 0.03 | -4.12% | -0.83 | 8.21 | 0.005 | 1162.1±39.4 | 1279.1±32.34 | -9.59% | -0.85 | 5.22 | 0.02 |  |  |  |  |  |  |
| R A post 10p | 2.37±0.028 | 2.45±0.023 | -3.32% | -0.84 | 4.62 | 0.03 | 1297.2±32.08 | 1391.3±26.33 | -7.00% | -0.85 | 5.09 | 0.03 |  |  |  |  |  |  |
| R Supp & Cing Eye Field | 2.92±0.035 | 3.05±0.029 | -4.36% | -0.90 | 8.04 | 0.005 | 1503.2±36.20 | 1598.3±29.71 | -6.27% | -0.82 | 4.08 | 0.05 |  |  |  |  |  |  |
|  | Cortical Thickness and Surface Area | | | | | | | | | | | | | | | | | |
| R A TE1 mid | 2.58±0.042 | 2.71±0.035 | -4.91% | -0.86 | 5.97 | 0.02 |  |  |  |  |  |  | 470.63±11.29 | 501.91±9.26 | -6.43% | -0.83 | 4.54 | 0.04 |

| **Morphometric Comparisons- nSZ vs HC** | | | | | | | | | | | | | | | | | | |
| --- | --- | --- | --- | --- | --- | --- | --- | --- | --- | --- | --- | --- | --- | --- | --- | --- | --- | --- |
|  | Cortical Thickness | | | | | | Volume | | | | | | Surface Area | | | | | |
| Region Name | nSZ Mean ± SD (mm) | HC Mean ± SD (mm) | Difference | Cohen’s D | F | Sig. | nSZ Mean ± SD (mm^3^) | HC Mean ± SD (mm^3^) | Difference | Cohen’s D | F | Sig. | nSZ Mean ± SD (mm^2^) | HC Mean ± SD (mm^2^) | Difference | Cohen’s D | F | Sig. |
|  | Volume and Surface Area | | | | | | | | | | | | | | | | | |
| L Area PGp |  |  |  |  |  |  | 1620.5±62.2 | 1790.7±43.11 | -9.98% | -0.85 | 4.99 | 0.03 | 472.61±16.88 | 515.63±11.70 | -8.71% | -0.83 | 4.33 | 0.04 |
| L Area PGs |  |  |  |  |  |  | 3001.8±79.08 | 3241.7±54.81 | -7.68% | -0.87 | 6.13 | 0.02 | 926.82±24.81 | 999.90±17.20 | -7.59% | -0.86 | 5.79 | 0.02 |
| R A TE1 mid |  |  |  |  |  |  | 1531.5±58.24 | 1688.6±40.36 | -9.76% | -0.84 | 4.85 | 0.03 | 461.16±13.92 | 501.82±9.65 | -8.44% | -0.86 | 5.69 | 0.02 |
|  | Cortical Thickness and Volume | | | | | | | | | | | | | | | | | |
| R A STSd ant | 2.72±0.037 | 2.85±0.025 | -4.67% | -0.90 | 8.14 | 0.01 | 1363.5±53.43 | 1500.7±37.03 | -9.58% | -0.83 | 4.39 | 0.04 |  |  |  |  |  |  |

| **Morphometric Comparisons- SZ vs nSZ** | | | | | | | | | | | | | | | | | | |
| --- | --- | --- | --- | --- | --- | --- | --- | --- | --- | --- | --- | --- | --- | --- | --- | --- | --- | --- |
|  | Cortical Thickness | | | | | | Volume | | | | | | Surface Area | | | | | |
| Region Name | SZ Mean ± SD (mm) | nSZ Mean ± SD (mm) | Difference | Cohen’s D | F | Sig. | SZ Mean ± SD (mm^3^) | nSZ Mean ± SD (mm^3^) | Difference | Cohen’s D | F | Sig. | SZ Mean ± SD (mm^2^) | nSZ Mean ± SD (mm^2^) | Difference | Cohen’s D | F | Sig. |
|  | Cortical Thickness, Volume, and Surface Area | | | | | | | | | | | | | | | | | |
| R Frontal Eye Field | 2.35±0.030 | 2.45±0.035 | -4.17% | -0.84 | 4.56 | 0.04 | 1174.9±37.38 | 1322.0±44.20 | -11.78% | -0.87 | 6.44 | 0.01 | 455.78±15.31 | 506.00±18.10 | -10.44% | -0.83 | 4.45 | 0.04 |
|  | Volume and Surface Area | | | | | | | | | | | | | | | | | |
| L Med Sup Temporal A |  |  |  |  |  |  | 594.80±21.27 | 522.40±25.15 | 12.96% | 0.84 | 4.79 | 0.03 | 291.32±8.10 | 265.79±9.57 | -9.17% | 0.82 | 4.11 | 0.05 |
| L TempPariet-Occip Junc 3 |  |  |  |  |  |  | 806.03±29.53 | 700.77±34.92 | 13.97% | 0.85 | 5.25 | 0.03 | 321.99±9.38 | 289.50±11.09 | 10.63 | 0.84 | 4.96 | 0.03 |
| R A Lat Intra-Parietal Dor |  |  |  |  |  |  | 414.18±22.30 | 491.23±26.37 | -17.01% | -0.84 | 4.94 | 0.03 | 206.87±8.22 | 233.36±9.72 | -12.03% | -0.83 | 4.29 | 0.04 |
| R A Lat Intra-Parietal ventr |  |  |  |  |  |  | 704.56±30.74 | 812.92±36.15 | -14.28% | -0.85 | 5.14 | 0.03 | 292.66±9.02 | 329.74±10.67 | -11.92% | -0.88 | 6.98 | 0.01 |
|  | Cortical Thickness and Volume | | | | | | | | | | | | | | | | | |
| L A 5L | 2.18±0.035 | 2.25±0.041 | -3.16% | -0.68 | 5.94 | 0.02 | 806.21±22.59 | 897.35±26.71 | -10.70% | -0.87 | 6.73 | 0.01 |  |  |  |  |  |  |
| L A 6 anterior | 2.51±0.024 | 2.58±0.028 | -2.75% | -0.80 | 4.58 | 0.04 | 2289.9±53.97 | 2460.5±63.81 | -7.18% | -0.82 | 4.13 | 0.05 |  |  |  |  |  |  |
| L A ant 47r | 2.69±0.040 | 2.83±0.047 | -5.07% | -0.85 | 5.39 | 0.02 | 2601.6±66.92 | 2860.4±79.13 | -9.48% | -0.87 | 6.19 | 0.02 |  |  |  |  |  |  |
| L Lat Intra-Parietal ventr | 2.17±0.031 | 2.30±0.036 | -5.82% | -0.89 | 8.13 | 0.006 | 785.66±28.55 | 917.18±33.56 | -15.45% | -0.90 | 8.77 | 0.004 |  |  |  |  |  |  |
| L Area OP1 | 2.83±0.043 | 2.97±0.050 | -4.83% | -0.83 | 4.31 | 0.04 | 798.79±21.10 | 878.29±24.94 | -9.48% | -0.86 | 5.87 | 0.02 |  |  |  |  |  |  |
| L Vent-Intra-Parietal Comp | 2.41±0.036 | 2.54±0.043 | -5.25% | -0.85 | 6.07 | 0.01 | 963.95±43.37 | 1156.7±51.28 | -18.18% | -0.90 | 8.17 | 0.006 |  |  |  |  |  |  |
| R infer 6-8 transitional A | 2.63±0.037 | 2.78±0.044 | -5.55% | -0.88 | 6.28 | 0.01 | 1108.8±62.33 | 1295.7±45.06 | -15.54% | -0.86 | 9.95 | 0.002 |  |  |  |  |  |  |

Supplementary table 4: Significant (Bonferroni corrected p<0.05) graph measures across threshold range. Reference Figure 1 in paper for better visualization.

| Threshold | 0.075 | 0.1 | 0.125 | 0.15 | 0.175 | 0.2 | 0.225 | 0.25 | 0.275 |
| --- | --- | --- | --- | --- | --- | --- | --- | --- | --- |
| Volume- Degree | | | | | | | | | |
| SZ | 242.01 | 206.86 | 174.27 | 144.61 | 118.00 | 95.07 | 74.89 | 58.11 | 44.89 |
| nSZ | 255.09 | 223.73 | 193.61 | 166.45 | 140.78 | 117.57 | 96.42 | 78.07 | 62.85 |
| HC | 215.08 | 174.10 | 137.31 | 106.41 | 80.08 | 58.92 | 43.13 | 30.84 | 21.80 |
| FEAP | 205.49 | 162.58 | 125.53 | 94.20 | 69.86 | 50.73 | 36.23 | 25.22 | 17.47 |
| Surface Area- Degree | | | | | | | | | |
| SZ | 246.77 | 213.67 | 182.37 | 152.61 | 126.51 | 103.76 | 83.47 | 66.44 | 52.91 |
| nSZ | 257.25 | 225.86 | 195.90 | 168.24 | 142.72 | 119.89 | 99.19 | 81.68 | 66.48 |
| HC | 219.94 | 180.32 | 145.33 | 114.74 | 88.90 | 67.90 | 51.14 | 38.41 | 28.65 |
| FEAP | 211.57 | 169.67 | 133.46 | 102.41 | 77.05 | 57.14 | 42.68 | 31.40 | 23.28 |
| Cortical Thickness- Degree | | | | | | | | | |
| SZ | 232.83 | 195.83 | 161.34 | 130.71 | 104.40 | 81.14 | 62.24 | 46.66 | 34.13 |
| nSZ | 253.85 | 221.57 | 192.13 | 164.23 | 138.42 | 115.33 | 94.84 | 76.62 | 61.58 |
| HC | 207.48 | 164.35 | 126.49 | 94.29 | 68.99 | 49.18 | 33.85 | 23.12 | 15.13 |
| FEAP | 198.60 | 153.52 | 116.27 | 85.96 | 61.10 | 42.24 | 28.72 | 18.72 | 12.09 |
| Volume- Clustering Coefficient | | | | | | | | | |
| SZ | 0.69 | 0.59 | 0.51 | 0.44 | 0.39 | 0.34 | 0.31 | 0.30 | 0.29 |
| nSZ | 0.72 | 0.64 | 0.56 | 0.49 | 0.43 | 0.38 | 0.34 | 0.31 | 0.29 |
| HC | 0.61 | 0.51 | 0.42 | 0.35 | 0.30 | 0.27 | 0.26 | 0.28 | 0.31 |
| FEAP | 0.59 | 0.48 | 0.39 | 0.33 | 0.29 | 0.27 | 0.28 | 0.30 | 0.37 |
| Surface Area- Clustering Coefficient | | | | | | | | | |
| SZ | 0.70 | 0.62 | 0.54 | 0.47 | 0.42 | 0.38 | 0.36 | 0.34 | 0.34 |
| nSZ | 0.73 | 0.64 | 0.57 | 0.50 | 0.44 | 0.40 | 0.36 | 0.34 | 0.32 |
| HC | 0.63 | 0.53 | 0.45 | 0.39 | 0.35 | 0.32 | 0.32 | 0.33 | 0.36 |
| FEAP | 0.61 | 0.50 | 0.42 | 0.36 | 0.32 | 0.31 | 0.32 | 0.35 | 0.40 |
| Cortical Thickness- Clustering Coefficient | | | | | | | | | |
| SZ | 0.66 | 0.56 | 0.47 | 0.40 | 0.34 | 0.29 | 0.26 | 0.25 | 0.25 |
| nSZ | 0.72 | 0.63 | 0.55 | 0.49 | 0.43 | 0.38 | 0.34 | 0.31 | 0.29 |
| HC | 0.59 | 0.48 | 0.38 | 0.31 | 0.26 | 0.24 | 0.23 | 0.26 | 0.33 |
| FEAP | 0.57 | 0.45 | 0.36 | 0.30 | 0.26 | 0.24 | 0.25 | 0.30 | 0.43 |
| Volume- Betweenness Centrality | | | | | | | | | |
| SZ | 115.98 | 151.14 | 183.73 | 213.39 | 240.00 | 262.93 | 283.15 | 300.61 | 321.88 |
| nSZ | 102.91 | 134.26 | 164.39 | 191.55 | 217.22 | 240.43 | 261.58 | 279.93 | 295.25 |
| HC | 142.92 | 183.90 | 220.69 | 251.59 | 277.92 | 299.25 | 321.82 | 376.52 | 469.77 |
| FEAP | 152.50 | 195.41 | 232.47 | 263.80 | 288.15 | 309.19 | 347.50 | 431.75 | 539.17 |
| Surface Area- Betweenness Centrality | | | | | | | | | |
| SZ | 111.22 | 144.32 | 175.63 | 205.39 | 231.49 | 254.23 | 274.53 | 292.02 | 310.97 |
| nSZ | 100.74 | 132.14 | 162.09 | 189.75 | 215.27 | 238.11 | 258.80 | 276.32 | 291.61 |
| HC | 138.06 | 177.68 | 212.66 | 243.26 | 269.09 | 290.27 | 312.32 | 352.63 | 425.15 |
| FEAP | 146.42 | 188.32 | 224.54 | 255.59 | 280.96 | 302.28 | 332.83 | 398.23 | 486.92 |
| Cortical Thickness- Betweenness Centrality | | | | | | | | | |
| SZ | 125.17 | 162.17 | 196.65 | 227.28 | 253.60 | 276.86 | 295.79 | 313.47 | 347.99 |
| nSZ | 104.15 | 136.42 | 165.87 | 193.77 | 219.58 | 242.66 | 263.16 | 281.38 | 296.61 |
| HC | 150.52 | 193.64 | 231.50 | 263.71 | 289.01 | 309.87 | 347.30 | 435.17 | 555.33 |
| FEAP | 159.40 | 204.47 | 241.73 | 272.04 | 296.96 | 322.69 | 384.79 | 502.01 | 644.92 |

Supplementary table 5: Number of modules at different threshold intervals across the σ range in all groups.

| **Threshold intervals** | **0.075** | **0.1** | **0.125** | **0.15** | **0.175** | **0.2** | **0.225** | **0.25** | **0.275** |
| --- | --- | --- | --- | --- | --- | --- | --- | --- | --- |
| **Number of modules in Volume SCN** | | | | | | | | | |
| **SZ** | 3 | 3 | 4 | 3 | 3 | 4 | 4 | 4 | 4 |
| **nSZ** | 4 | 4 | 4 | 4 | 4 | 4 | 4 | 4 | 4 |
| **FEAP** | 3 | 4 | 4 | 4 | 4 | 4 | 4 | 4 | 6 |
| **HC** | 4 | 3 | 4 | 4 | 4 | 4 | 4 | 4 | 6 |
| **Number of modules in Surface Area SCN** | | | | | | | | | |
| **SZ** | 3 | 4 | 4 | 4 | 4 | 4 | 4 | 4 | 4 |
| **nSZ** | 3 | 3 | 4 | 4 | 4 | 4 | 4 | 5 | 4 |
| **FEAP** | 4 | 4 | 4 | 4 | 4 | 5 | 4 | 5 | 6 |
| **HC** | 3 | 4 | 4 | 4 | 4 | 4 | 5 | 6 | 6 |
| **Number of modules in Cortical Thickness SCN** | | | | | | | | | |
| **SZ** | 4 | 4 | 4 | 4 | 4 | 4 | 4 | 4 | 4 |
| **nSZ** | 4 | 4 | 4 | 4 | 4 | 4 | 4 | 4 | 4 |
| **FEAP** | 4 | 3 | 4 | 4 | 4 | 4 | 4 | 5 | 8 |
| **HC** | 4 | 4 | 4 | 4 | 4 | 4 | 4 | 5 | 7 |

Supplemental Figure 1: Betweenness and Eigenvector Centrality Removal Distribution Throughout Brain. Normalized betweenness centrality (left) and eigenvector centrality (right) distribution throughout all brain regions for all groups in the cortical thickness SCN. A region colored in dark red represents the highest measure and would be removed first in the removal simulations, and dark blue represents the lowest measure and would be removed last. Gray regions, NA on the color bar, are subcortical and are not in the analysis.

Supplemental Figure 2: (A) Attack based on eigenvector centrality across the small-worldness range for the cortical thickness SCNs for FEAP (bottom) and HC (top). (B) Attack on betweenness centrality ranked nodes for SZ and nSZ and their age/sex matched HC groups for the cortical thickness SCN. (C) Attack on eigenvector centrality ranked nodes for SZ and nSZ and their age/sex matched HC groups for the cortical thickness.
